# Inhibition of SOX2 induces cell apoptosis and G1/S arrest in Ewing’s sarcoma through the PI3K/Akt pathway

**DOI:** 10.1186/s13046-016-0321-3

**Published:** 2016-03-11

**Authors:** Chongmin Ren, Tingting Ren, Kang Yang, Shidong Wang, Xing Bao, Fan Zhang, Wei Guo

**Affiliations:** Musculoskeletal Tumor Center, Peking University People’s Hospital, No. 11 Xizhimen South Street, Beijing, 100044 People’s Republic of China; Beijing Key Laboratory of Musculoskeletal Tumor, Beijing, 100044 People’s Republic of China

**Keywords:** Ewing’s sarcoma, SOX2, Cell cycle, Apoptotsis, PI3K/Akt signaling

## Abstract

**Background:**

Ewing’s sarcoma is an aggressive bone and soft tissue tumor with a high incidence in children and adolescents. Due to its high malignancy and poor prognosis, identification of novel biomarkers for intervention therapies is necessary to improve outcome. The *EWS/FLI1* fusion gene is a characteristic of Ewing’s sarcoma in most cases. Sex determining region Y-box 2 (*SOX2*) is a primary target of *EWS/FLI1*. It has been identified as an oncogene and linked to apoptotic resistance in several types of cancer. However, its role and regulatory mechanisms in Ewing’s sarcoma are largely unknown.

**Methods:**

We systematically investigated the role of SOX2 in Ewing’s sarcoma cell lines, human tissue samples and xenograft models. The expression of SOX2 was detected in Ewing’s sarcoma samples by WB and IHC. siRNAs were used to knockdown EWS/FLI1 and SOX2 in A673 and RD-ES cell lines with the efficiencies tested by qRT-PCR and WB. The effect of SOX2 on cell cycle and apoptosis was determined by Flow cytometric and TUNEL assays. Akt overexpression was performed with plasmid. The protein expression of the corresponding factors was examined by WB analysis. Inhibition of SOX2 in vivo was performed by siRNA against SOX2 in xenograft models, and the protein expression of the regulators testified in vitro was examined in xenograft tumors by IHC and WB.

**Results:**

The results confirmed that SOX2 was highly expressed in Ewing’s sarcoma and was the target of EWS/FLI1. SOX2 advanced Ewing’s sarcoma cell survival and proliferation by regulating p21, p27 and cyclin-E to facilitate G1/S phase transition and mediating caspase-3, PARP *via* both extrinsic (Fas and caspase-8) and intrinsic (caspase-9, Bad, Bcl-2 and XIAP) apoptotic pathways to restrain cell apoptotsis. Additionally, SOX2 regulated the cell-cycle progression and apoptosis *via* activation of the PI3K/Akt signaling pathway. The mechanisms were proved both in vitro and in vivo.

**Conclusions:**

The results demonstrate that SOX2 played a central role in promoting Ewing’s sarcoma cell proliferation in vitro and in vivo with the underlying mechanisms expounded. These findings suggest that SOX2 may serve as a potential biomarker for targeted intervention in Ewing’s sarcoma.

## Background

Ewing’s sarcoma is the second most common bone and soft tissue malignancy after osteosarcoma with a high incidence in children and adolescents [1, 2]. It is associated with rapid progression, a tendency to metastasize and shows a high rate of recurrence after resection. Despite advances in combinational therapies, the prognosis of patients with Ewing’s sarcoma remains poor with low survival rates [1, 3]. Therefore, identification of novel biomarkers for target intervention is essential in order to improve treatment outcomes.

Ewing’s sarcoma is characterized by unique chromosomal translocations; the most common is t(11;22) (q24;q12) generating the *EWS/FLI1* fusion gene that accounts for 85 % of all cases [4, 5]. *EWS/FLI1* encodes a chimeric protein consisting of the transcriptional activation domain of EWS and the DNA-binding domain of FLI1 [6, 7]. Recombination gives rise to an aberrant transcriptional factor, believed to be implicated in the origin of ESFT [8]. Transfection of EWS/FLI1 into mesenchymal stem cells (MSC) revealed that EFTS cancer stem cells (CSC) express genes associated with embryonic stem cell (ESC), including *SOX2*, *OCT4* and *NANOG* [9, 10]. Of these, *SOX2* (sex determining region Y-box 2) was identified as a key EWS/FLI1 target gene and shown to participate in the progression of ESFT [11].

SOX2 is a critical transcriptional factor for self-renewal and maintenance of undifferentiated ESCs, and was initially selected for deriving induced pluripotent stem (iPS) cells [12, 13]. Since its close association with CSCs [11, 14–22], SOX2 has been reported as being overexpressed in many aggressive tumors [23–29]. Its role as an oncogene in malignant transformation has been confirmed in multiple studies showing that it could promote tumor cell growth [11, 15, 17, 22–28] and advance tumorigenesis [11, 15, 19–21, 24]. SOX2 has been linked to apoptotic resistance [21, 23, 24, 26–28] and shown to facilitate cell cycle progression [22–25, 27] in certain types of cancers. For example, repression of SOX2 was found to induce cell apoptosis *via* cleavage of caspase-3 and activation of specific pro-apoptotic factors [23, 24, 26], and inhibit G1/S transition by regulating cyclin E in prostate cancer [23, 27] and cyclin D1 in breast cancer [25]. In addition, SOX2 was identified as a regulatory factor in several key signaling pathways associated with tumor progression, including the Akt, Wnt and MAPK pathways [16, 19, 20, 23, 25–29]. Although SOX2 has been strongly implicated with Ewing’s sarcoma cell proliferation and tumorigenesis, the specific regulatory mechanisms remain unclear [11]. Therefore, the purpose of this study was to determine the role of SOX2 in the progression of Ewing’s sarcoma and elucidate the underlying mechanisms.

## Methods

Unless otherwise stated, all solutions and materials were standard analytical grade laboratory stocks. Experiments were repeated as indicated in the figure legends for statistical analysis.

### Tissue specimens, cell lines and culture

Formalin-fixed paraffin-embedded (FFPE) specimens of Ewing’s sarcomas and normal soft tissues around bones were acquired from the Musculoskeletal Tumor Center, Peking University People’s Hospital, Beijing, China. All patients provided written informed consent and the study was approved by the center’s Ethics Committee.

Human Ewing’s sarcoma cell lines A673 and RD-ES (American Type Culture Collection; ATCC; Rockville, MD, USA) were maintained in a humidified atmosphere of 5 % CO_2_ at 37 °C in DMEM or RPMI-1640 medium, respectively, supplemented with 10 % fetal bovine serum (FBS; Hyclone, Logan, UT, USA) and streptomycin/penicillin antibiotics.

### Immunohistochemistry

Tissue specimens were sectioned (4 μm thickness) and dewaxed in dimethylbenzene before being rehydrated through an increasing ethanol gradient. The sections were rinsed in PBS and incubated in 3 % hydrogen peroxide for 15 min at room temperature. They were blocked in 10 % goat serum for 30 min prior to incubation with rabbit polyclonal antibodies (1:100 dilution) overnight at 4 °C followed by incubation with secondary antibody for 1 h at room temperature. Diaminobenzidine was added as a chromogen and the sections were counterstained with hematoxylin. Positive staining was defined as >10 % of cells appearing as brown granules. Antibodies against SOX2 (3579), p-Akt (4060), cyclin-E (4136) and cleaved-caspase-3 (9661) for immunohistochemistry were purchased from Cell Signaling Technology (Beverly, MA, USA).

### Knockdown of genes with siRNAs and overexpression of Akt with plasmids

SiRNAs were purchased from OriGene Technologies (Rockville, MD, USA). Knockdown of genes was performed using siRNAs against *EWS/FLI1* (siEF [30, 31] and siEF-bp [30, 32]) and *SOX2* (siSOX2-#1 and siSOX2-#2). A stable non-specific siRNA (siNC) was used as a negative control. The sequences were as follows: siEF, 5′-GUACCCUUCUGACAUCUCCTT-3′(sense) and 5′-GGAGAUGUCAGAAGGGUACTT-3′(antisense); siEF-bp, 5′-GCAGAACCCUUCUUAUGACTT-3′(sense) and 5′-GUCAUAAGAAGGGUUCUGCTT-3′(antisense); siSOX2-#1, 5′-CCAUGGGUUCGGUGGUCAATT-3′(sense) and 5′-UUGACCACCGAACCCAUGGTT-3′(antisense); siSOX2-#2, 5′-GGACAUGAUCAGCAUGUAUTT-3′(sense) and 5′-AUACAUGCUGAUCAUGUCCTT-3′(antisense); and siNC, 5′-UUCUCCGAACGUGUCACGUTT-3′(sense) and 5′-ACGUGACACGUUCGGAGAATT-3′(antisense). A673 and RD-ES cells were transfected at 50–60 % confluence with 200 nmol/L siRNAs using Lipofectamine 2000 Transfection Kit (Life Technologies-Invitrogen; Carlsbad, CA, USA) according to the manufacturer’s protocol.

Overexpression of AKT was carried out using a GV219 plasmid (Genechem, Shanghai, China) carrying the AKT1 cDNA insert (NM_005163); an empty vector was used as a negative control. Plasmids were transfected into cells at 90 % confluence using the Lipofectamine 2000 Transfection Kit according to the manufacturer’s protocol.

After 48 h transfection, cells were collected for further analyses.

### Real-time quantitative reverse transcription PCR (qRT-PCR)

Total RNA was extracted using a PureLink RNA Mini Kit (Ambion, Life Technologies). CDNA was synthesized with purified RNA using SuperScript III First-Strand Synthesis SuperMix (Invitrogen). Amplification was performed using primers with SYBR GreenER qPCR SuperMix Universal (Invitrogen). GAPDH was used as a control. All protocols were performed according to the manufactures’ instructions. The primer sequences were as follows: EWS/FLI1, 5′-CGACTAGTTATGATCAGAGCAGT-3′(forward) and 5′-CCGTTGCTCTGTATTCTTACTGA-3′ (reverse); SOX2, 5′-CAGGAGTTGTCAAGGCAGAGA-3′ (forward) and 5′-CAGGAGTTGTCAAGGCAGAGA-3′ (reverse); GAPDH, 5′-ACAACTTTGGTATCGTGGAAGG-3′ (forward) and 5′-GCCATCACGCCACAGTTTC-3′ (reverse). Reactions were performed for 2 min at 50 °C, 10 min at 95 °C, followed by 40 cycles of 15 s at 95 °C and finally 30 s at 56 °C and 30 s at 72 °C.

### Western blot assays and antibodies

Tissue samples and cells were lysed using RIPA lysis buffer and centrifuged before collecting the supernatants. Protein concentrations was evaluated by UV absorbance (280 nm) and samples containing equal amounts of protein were separated on 8–12 % SDS-polyacrylamide gels using a NuPAGE system (Invitrogen). Following separation, the proteins electrophoretically transferred onto PVDF membranes and blocked in non-fat milk for 1 h. The membranes were then incubated with primary antibodies overnight at 4 °C followed by incubation with HRP-conjugated secondary antibodies for 1.5 h at room temperature. Immunoreactive bands were visualized and quantified using an electrophoresis image analysis system (Bio-Rad; Hercules, CA, USA).

Antibodies against SOX2 (3579, 1:1000), PI3K (11189, 1:500), p-Akt (9271, 1:500), Fas (4233, 1:500), Bad (9239, 1:500), Bcl-2 (2872, 1:1000), XIAP (2042, 1:1000), Caspase-8 (9746, 1:500), Caspase-9 (9508, 1:1000), Caspase-3 (9662, 1:1000), PARP (9542, 1:1000), Cyclin-E (4129, 1:500) and Cyclin-D1 (2922, 1:1000) were purchased from Cell Signaling Technology (Beverly, MA, USA). Anti-Fli1 (sc-356, 1:500), anti-Akt (sc-8312, 1:500), anit-p21 (sc-271532, 1:500), anti-p27 (sc-71813, 1:500) and GAPDH (sc-365062 1:1000) were purchased from Santa Cruz Biotechnology (Santa Cruz, CA, USA).

### Cell viability and proliferation assays

The MTT cell viability assay was performed by plating transfected cells into 96-well plates at a density of 3 × 10^3^ cells/well. The cells were incubated for 72 h prior to adding MTT solution (Sigma-Aldrich; St. Louis, MO, USA) and cultured for a further 4 h. The cells were lysed with dimethyl sulfoxide (DMSO) and absorbance was measured at 490 nm using a microplate reader (Molecular Devices; Sunnyvale, CA, USA).

The colony formation assay was performed by plating transfected cells at logarithmic growth phase into 6-well plates at a concentration of 1 × 10^3^ cells/well. Colonies were allowed to form for 7 days before the cells were fixed with ice-cold methanol and stained with crystal violet solution. The numbers of colonies were counted under a microscope: a colony was defined as a group containing ≥10 cells.

### Flow cytometric assays for cell cycle distribution and apoptosis

The cell cycle assay was performed by fixing the cells in 70 % ethanol at 4 °C overnight and treated with 0.2 % Triton X-100 and RNase before being stained with Propidium Iodide (PI) for 30 min in darkness. The cells were analyzed using an Accuri C6 flow cytometer (Accuri Cytometers Inc.; Ann Arbor, MI, USA) and the percentages of cells at each phase of cell cycle were determined.

The cell apoptosis assay was performed using an Annexin-V/FITC Kit (BD Biosciences; San Jose, CA, USA). Transfected-cells were collected and washed in ice-cold PBS before being stained with Annexin V and PI solution for 15 min in darkness. The ratios of apoptotic cells were determined using an Accuri C6 flow cytometer.

Cell cycle and apoptosis were analyzed by the flow cytometer assays 48 h after transfection.

### TUNEL assay of cell apoptosis

Cells were seeded onto slides and cultured before being treated with appropriate siRNAs. The cells were then fixed in 4 % paraformaldehyde for 60 min and permeabilized with 0.1 % Triton X-100 on ice for 2 min. Cell apoptosis was determined using a TUNEL Apoptosis Assay Kit (Roche Applied Science; Mannheim, Germany) according to the manufacturer′s instructions 48 h post transfection. Briefly, TUNEL (TdT-mediated dUTP nick end labeling) reaction mixture was added to the cells and the slides were rinsed in PBS before being incubated in a humidified chamber at 37 °C for 60 min in darkness. Anti-fluorescence quenching solution was added before examining the cells under a confocal laser-scanning microscope (TCS SP5) at an excitation wavelength of 450–500 nm and an emission wavelength of 515–565 nm (green fluorescence) in order to evaluate the proportions of apoptotic cells.

### Xenograft tumorigenicity assays

BALB/c athymic mice at 6–8 weeks old were acquired from Vital River (Beijing, China) and kept according to animal welfare guidelines of Peking University People’s Hospital. Each mouse was injected in the right flank with 3 × 10^6^ A673 Ewing’s sarcoma cells. When the xenograft tumors were palpable, the mice were randomly divided into three groups. For each group, intratumoral injection with siSOX2, siNC or glucose was performed once every 2 days. Five microgram siSOX2 or siNC was mixed with 8 μl transfection reagent (Entranster-in vivo; Engreen, Beijing, China) and hydrated with 10 % glucose injection at a concentration of 50 μg/ml for each injection. The tumor volumes were calculated ([length × width^2^]/2) every 3 days and the data were plotted for comparison. After 30 days, the mice executed humanely and the tumors were excised and photographed. Xenograft tissue samples were prepared for subsequent IHC and Western blot assays.

### Statistical analysis

All the statistical analyses were performed using SPSS v.19.0 software (SPSS; Chicago, IL, USA). Independent two-sample t-tests were used to compare differences between two groups. One-way analysis of variance (ANOVA) with the multiple comparison test was used to analyze differences between three or more groups. All data were presented as mean ± standard deviation based on three independent experiments. A *P*-value <0.05 was considered statistically significant.

## Results

### SOX2 was a downstream target of EWS/FLI1 in Ewing’s sarcoma cells

**Fig. 1:**
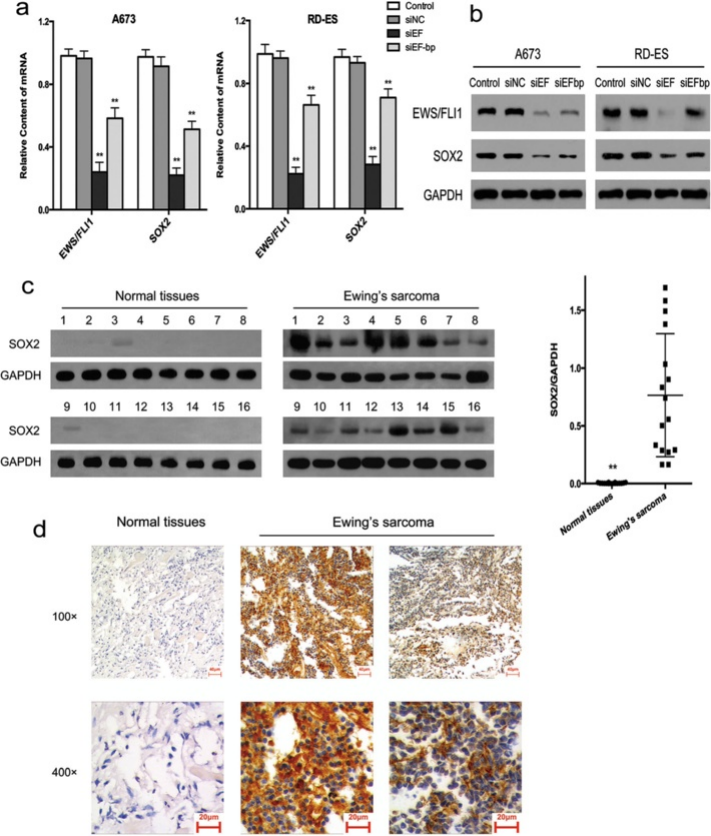
SOX2 was regulated by EWS/FLI1 and highly expressed in Ewing’s sarcoma. **a**, and **b** Expression of SOX2 was significantly decreased by EWS/FLI1 knockdown in A673 and RD-ES cells in terms of both on mRNA and protein levels. Data are representative of three independent experiments. **c** SOX2 was expressed in Ewing’s sarcoma but not in normal tissues around bones examined by WB analysis. **d** Representative images of IHC staining of SOX2 in normal tissues and Ewing’s sarcoma. All data are presented as means ± S.D. **P < 0.001

In order to confirm the relationship between SOX2 and EWS/FLI1 in Ewing’s sarcoma, two small interfering RNAs (siRNAs) were used to knockdown *EWS/FLI1* in A673 and RD-ES cells. The knockdown efficiencies were determined for mRNA and protein expressions by real-time quantitative reverse transcription PCR (qRT-PCR) and Western blot assays, respectively. The results showed that expression of EWS/FLI1 was downregulated at both mRNA and protein levels in the transfected cells, with a concurrent reduction in the expression of SOX2 (Fig. 1a and b; *P* < 0.001). These results confirmed that SOX2 was a downstream regulatory target of EWS/FLI1 in Ewing’s sarcoma cells.

### SOX2 was highly expressed in Ewing’s sarcoma tissue samples

Expressions of SOX2 protein in human tissue samples acquired from Ewing’s sarcomas and normal tissues around bones were determined by Western blot and immunohistochemistry (IHC) assays. Western blotting showed that protein expression of SOX2 was markedly higher in Ewing’s sarcoma samples compared to levels in normal tissues (Fig. 1c; *P* < 0.001). Similar results were obtained by IHC, as indicated by positive staining in sarcoma tissues and negative staining in normal tissues (Fig. 1d). These results implied that SOX2 played a key role in Ewing’s sarcoma.

### Knockdown of SOX2 inhibited cell viability and growth in Ewing’s sarcoma in vitro

**Fig. 2:**
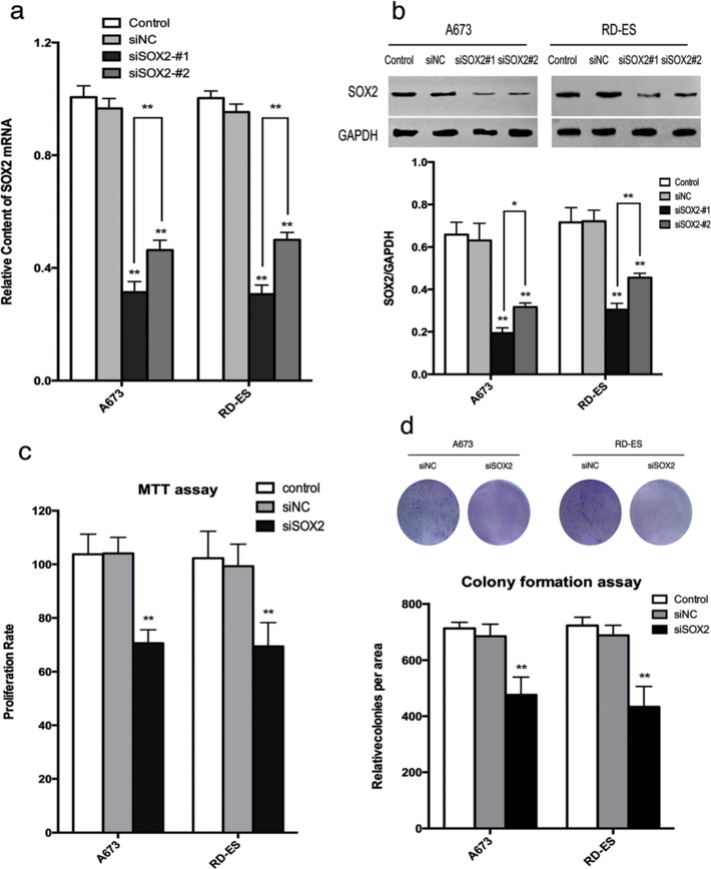
SOX2 knockdown by siRNA inhibited the growth capacity of Ewing’s sarcoma cells. **a**, and **b** The mRNA and protein expression levels of SOX2 were significantly repressed by the transfection of SOX2 siRNA in both A673 and RD-ES cells. **c** The cell viabilities were inhibited in both cell lines after SOX2 knockdown according to the MTT assay. **d** SOX2 inhibition resulted in a decreased colony formation capacity in both A673 and RD-ES cell lines. Representative pictures were displayed and the colonies per area were shown as data in bars. All data are presented as means ± S.D. *P < 0.01, **P < 0.001

In order to determine whether overexpression of SOX2 advanced the growth capacity of Ewing’s sarcoma cells, two different siRNAs against SOX2 were used to knockdown expression of SOX2 in A673 and RD-ES cell lines. Expression levels were compared to those in controls that had been treated without siRNA and negative controls (siNC). Both SOX2 mRNA and protein expressions were significantly downregulated in the silenced cells (siSOX2-#1 and siSOX2-#2) compared to control cells (Fig. 2a and b, respectively; *P* < 0.001). The most effective siRNA (siSOX2-#1;*P* < 0.01 to *P* <0.001) was the selected for use in subsequent experiments.

The MTT cell viability assays showed that knockdown of SOX2 reduced cell viability by approximately 30 % in both cell lines compared to controls (Fig. 2c; *P* < 0.001). Furthermore, knockdown of SOX2 suppressed the colony forming ability of the cell lines (Fig. 2d; *P* < 0.001).

### Suppression of SOX2 induced G1/S cell cycle arrest by targeting cyclin-E, p21 and p27

**Fig. 3:**
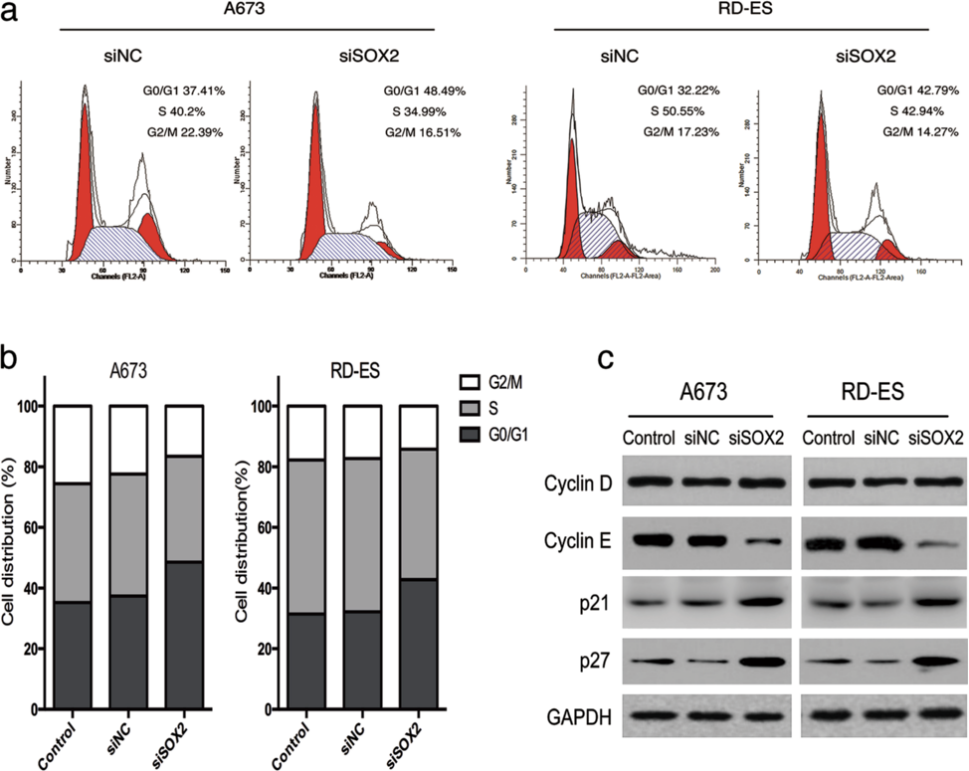
SOX2 inhibition induced G1/S cell cycle arrest. **a**, and **b** The cell cycle was assayed by flow cytometry. The percentages of cell cycle phases were displayed in bar charts. siSOX2 transfection resulted in a significant increase of the cells proportion in the G0/G1 phases of A673 and RD-ES cells. **c** SOX2 inhibition amplified the expression of p21 and p27, and repressed cyclin-E, but had no effect on cyclin-D expression in Ewing’s sarcoma cells according to assayed by WB analyses. The data are representative and selected from one of three independent experiments

The effect of SOX2 knockdown on cell cycle distribution in Ewing’s sarcoma cells was investigated by flow cytometry in A673 and RD-ES cell lines as an indication of proliferative capacity. The results showed a significant increase in the percentage of SOX2-knockdown cells at G0/G1 phase compared to control cells, with a concurrent decrease in the proportion of cells at S and G2/M phases (Fig. 3a and b; *P* < 0.001). This indicated that silencing SOX2 induced cell cycle arrest at G1/S phase.

To explore the potential mechanism, the protein expressions of four critical regulators of G1/S transition (cyclin-D1, cyclin-E, p21^cip1^ and p27^kip1^) [33–36] were analyzed by Western blotting. Expressions of cyclin-dependent kinase (CKD) inhibitors p21 and p27 were markedly increased after knockdown of SOX2 compared to control cells; expression of cyclin-E was decreased; however, there was no significant change in expression of cyclin-D1 (Fig. 3c). Cyclin-E binds CDK2 to induce G1/S transition [33, 34], whereas p21 and p27 repress cyclin-E/CDK2 complex to inhibit the processes [35, 36]; therefore these results indicated that inhibition of SOX2 in Ewing’s sarcoma cells was induced by repression of cyclin-E and activation of p21 and p27.

### Inhibition of SOX2 promoted cell apoptosis in Ewing’s sarcoma *via* activation of extrinsic and intrinsic apoptotic pathways

**Fig. 4:**
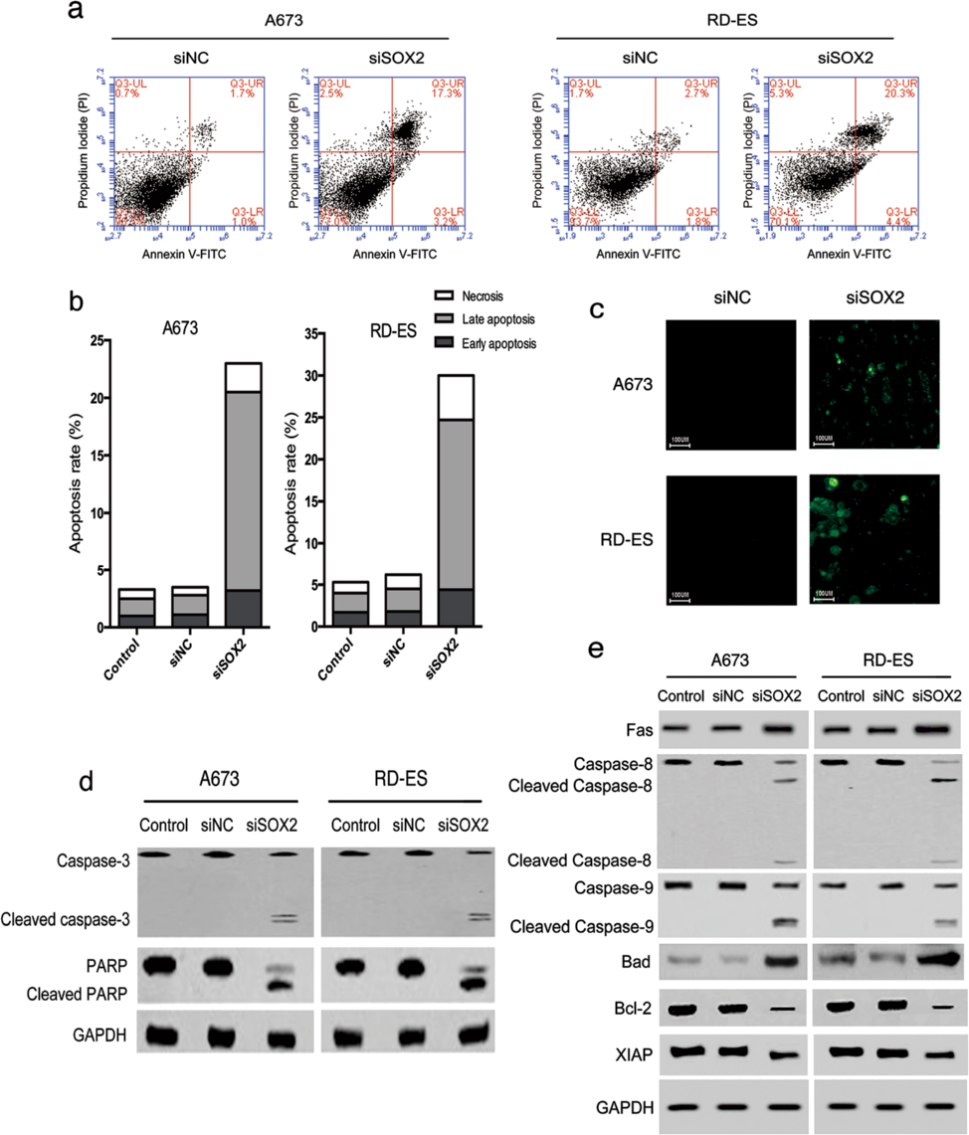
SOX2 knockdown promoted the apoptosis of Ewing’s sarcoma cells by activating the extrinsic and intrinsic apoptotic pathways. **a**, and **b** Cell apoptosis analysis was performed by flow cytometry. The *bar* charts showed the significant increases in the early and late apoptotic indexes of A673 and RD-ES cells transfected with siSOX2. **c** Cell apoptosis of A673 and RD-ES cells treated with siSOX2 were evaluated by the TUNEL assay. **d** Cleavage of caspase-3 and PARP were enhanced in Ewing’s sarcoma cells transfected with siSOX2, as assayed by WB. **e** The proteins of the cell death receptor and mitochondrial apoptosis pathways were examined by WB assays in cells under SOX2 knockdown conditions performed. SOX2 inhibition amplified pro-apoptotic regulators, such as Fas and Bad, repressed anti-apoptotic mediators, such as Bcl-2 and XIAP, and activated caspases-8 and caspase-9. The data are representative of three independent experiments

To investigate whether knockdown of SOX2 induced apoptosis in Ewing’s sarcoma cells, flow cytometric and TUNEL assays were performed in A673 and RD-ES cell lines. Flow cytometric analysis showed that the proportions of early and late apoptotic cells were significantly increased by silencing SOX2 in both cell lines compared to those in controls (Fig. 4a and b; *P* < 0.001). Similarly, the TUNEL assay showed an increase in apoptotic cells, as evidenced by enhanced fluorescence, in both cell lines following knockdown of SOX2, confirming that inhibition of SOX2 induced apoptosis in Ewing’s sarcoma cells (Fig. 4c).

To elucidate the potential mechanism, two key regulators of cell apoptosis, caspase-3 and poly ADP ribose polymerase (PARP), were examined by Western blotting. PARP plays a central role in DNA repair; however, cleavage of PARP results in loss-of-function and can be induced following activation of caspase-3 *via* cleavage [37]. The analyses showed that transfection with siSOX2 resulted in marked increases in cleavages of caspase-3 and PARP following compared to the levels in control cells (Fig. 4d).

To further explore the molecular mechanism underlying, Western blotting was used to determine the protein expressions of specific apoptotic mediators (caspase-8, caspase-9, Fas, Bad, Bcl-2 and XIAP). Caspase-3 is the downstream target of caspase-8 and caspase-9 [38]; caspase-8 is a key protein in extrinsic apoptotic pathways and is stimulated by cell surface death receptors, such as Fas [38, 39]; caspase-9 is a key protein in intrinsic apoptotic pathways activated by mitochondrial dysfunction, which involved Bcl-2 family proteins, like Bad and Bcl-2 [38, 40]; and XIAP is an anti-apoptotic protein inhibiting caspase-3 and −9 [38, 41]. The results showed that caspases-8 and −9 were fully activated, as evidenced by complete cleavage of both proteins, with concurrent elevation of pro-apoptotic proteins Bad and Fas; whereas expressions of anti-apoptotic proteins Bcl-2 and XIAP were substantially decreased compared to controls (Fig. 4e). These results indicated that inhibition of SOX2 induced activation of both extrinsic cell death receptor and intrinsic mitochondrial apoptotic pathways, leading to apoptosis in Ewing’s sarcoma cells.

### SOX2 regulated cell-cycle progression and apoptosis in Ewing’s sarcoma *via* activation of the PI3K/Akt signaling pathway

The PI3K/Akt signaling pathway plays an essential role in cell survival and cell growth *via* direct or indirect regulation of apoptotic factors and cell cycle regulators, including those identified as targets of SOX2 in our previous analyses (caspase-9, Bad, Fas, p21, p27) [42–45]. This suggested a functional relationship between SOX2 and the PI3K/Akt signaling pathway in Ewing’s sarcoma cells.

**Fig. 5:**
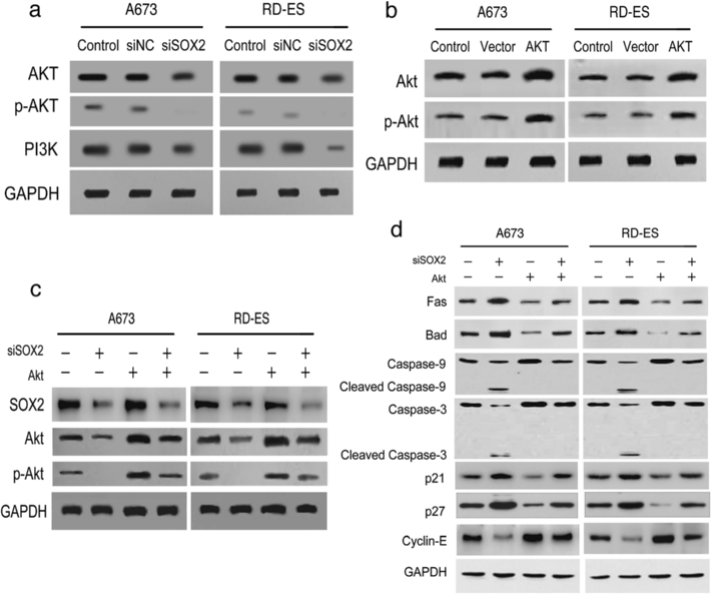
SOX2 was found to regulate cell cycle and apoptosis of Ewing’s sarcoma cells *via* the PI3K/Akt signaling pathway. **a** WB assay showed SOX2 knockdown resulted in decreased expressions of PI3K and Akt and the loss of p-Akt in A673 and RD-ES cells. **b** Overexpression of Akt was evaluated by WB analysis. **c** Four groups were designed for Ewing’s sarcoma cells as mock control, SOX2 knockdown, Akt overexpression and the contransfection of siSOX2 and Akt plasmids. Expression levels of SOX2, Akt and p-Akt of the four groups were examined by WB. **d** Further investigation of the proteins targeted by SOX2 and Akt involved in the Ewing’s sarcoma cells apoptosis and G1/S transition, including Fas, Bad, caspase-9, caspase-3, p21, p27 and cyclin-E, were analyzed by WB in the four designed groups. Representative data from one of three independent experiments are exhibited in (**a**–**d**)

To verify this supposition, the expressions of PI3K, Akt and p-Akt proteins in A673 and RD-ES cells following knockdown of SOX2 were examined by Western blotting. The results showed marked reductions in PI3K and Akt protein expression levels with an almost complete loss of p-Akt expression compared to the controls (Fig. 5a). These findings confirmed that SOX2 was an upstream mediator in the PI3K/AKT signaling pathway in Ewing’s sarcoma cells. To further demonstrate that SOX2 was implicated in the proliferation and progression of Ewing’s sarcoma, we reactivated the PI3K/Akt pathway after knockdown of SOX2 by overexpressing Akt using plasmids. Western blotting was performed to confirm that the expressions of Akt and p-Akt proteins were raised by Akt overexpression (Fig. 5b). Four test groups were prepared: untreated controls (−/−), SOX2 knockdown (+/−), Akt overexpression (−/+), and cotransfection of siSOX2 + Akt plasmid (+/+). Knockdown of SOX2 resulted in complete loss of p-Akt protein expression, as previously demonstrated; however, in the contransfected group, this was counteracted by overexpression of Akt resulting in the recovery of p-Akt to its untreated levels. The Akt overexpression group showed increases of Akt and p-Akt protein but no significant changes of SOX2 protein expression (Fig. 5c).

Inhibition of Akt phosphorylation through knockdown of SOX2 was found to elevate protein expressions of pro-apoptotic factors Bad and Fas, induce cleavage of caspase-9 and caspase-3, promote expression of p21 and p27, and suppress expression of cyclin-E compared to the corresponding levels in untreated controls. Recovery of p-Akt through overexpression of Akt negated these effects in the cotransfection group (Fig. 5d).

These findings indicated that regulation of apoptotic and G1/S transitional factors in Ewing’s sarcoma cells were induced by SOX2 *via* activation of the PI3K/Akt pathway.

### Suppression of SOX2 inhibited tumorigenesis in Ewing’s sarcoma in vivo

**Fig. 6:**
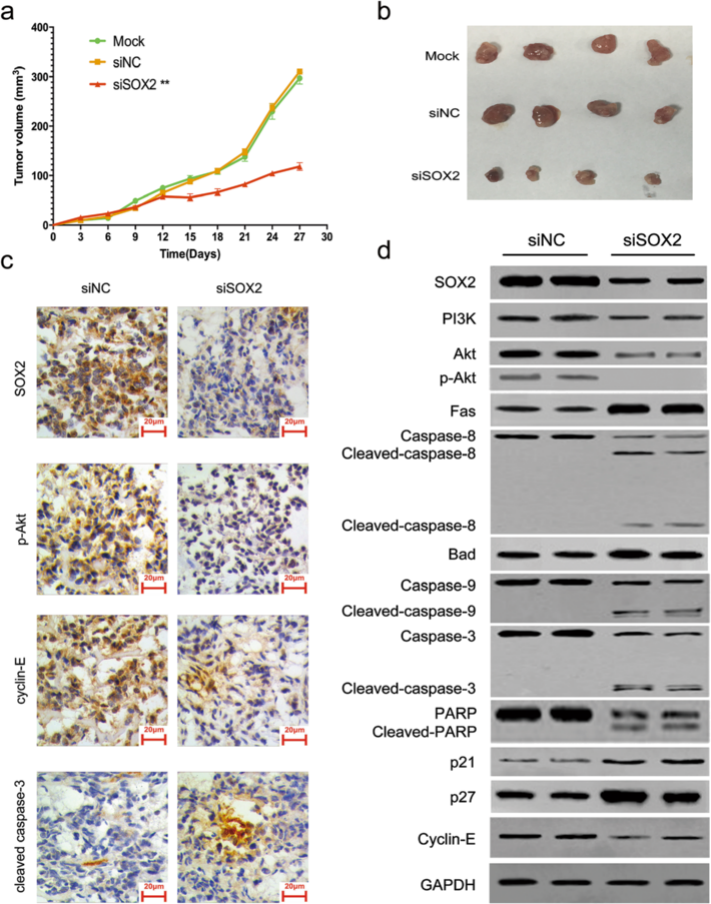
SOX2 inhibition suppressed Ewing’s sarcoma growth in vivo. **a** Tumor growth was significantly repressed by siSOX2 intratumoral injection as shown in the curve graph (Data are presented as the mean ± S.D. **P < 0.001). **b** SOX2 inhibition substantially decreased xenograft tumor volume, shown as representative images exhibited. **c** Representative images of IHC analysis demonstrated the significant repression of SOX2, p-Akt and cyclin-E, and the enhancement of cleaved-caspase-3 in tumor samples treated with siSOX2. **d** Variations of the upstream regulators, such as SOX2, PI3K, Akt and p-Akt, the downstream anti-survival of proteins, such as Fas, caspase-8, Bad, caspase-9, caspase-3, p21 and p27, and the pro-proliferation factors, such as PARP and cyclin-E, were all followed the same trends described as in vitro, as analyzed by WB. The data are representative of three independent experiments

To confirm our in vitro findings in vivo, Ewing’s sarcoma xenograft models were established in mice. The mice received intramural injections of siSOX2 (siSOX2 group), glucose (mock control group) or siNC (negative control group). The results showed that rate of tumor growth and the sizes of the tumors were significantly lower in the siSOX2 group compared to those in the control groups (Fig. 6a and b; *P* < 0.001). These findings confirmed that inhibition of SOX2 could significantly repress tumorigenesis in Ewing’s sarcoma in vivo.

In order to determine whether the proteins identified as influencing cell cycle transition and apoptosis in the in vitro experiments were observed in vivo, IHC and Western blot assays were performed using tissue samples from the xenograft models. As observed in vitro, the results confirmed that SOX2 regulated apoptotic and cell cycle factors *via* mediation of PI3K/Akt signaling: IHC showed that positive staining for pro-proliferation factors (SOX2, p-Akt and cyclin-E) was lower in xenograft tissues from the siSOX2 group compared to those from the siNC group. In contrast, positive staining for pro-apoptotic cleaved-caspase-3 was higher in xenograft samples from the siSOX2 group compared to that in the siNC group (Fig. 6c). Western blotting revealed that xenografts treated with siSOX2 had reduced expressions of PI3K and Akt proteins and almost no expression of p-Akt compared to the corresponding levels in the siNC group. This confirmed that silencing SOX2 inhibited PI3K/Akt signaling in vivo. Similarly, silencing SOX2 elevated protein expressions of pro-apoptotic factors Fas and cleaved-caspase-8 (mediators in the cell death receptor pathway), Bad and cleaved-caspase-9 (mediators in the mitochondrial apoptotic pathway), as well as cleaved-caspase-3 (activated by the cell death receptor and mitochondrial apoptotic pathway) and cleaved-PARP, demonstrating that SOX2 inhibition induced cell apoptosis. In addition, protein expressions of cell cycle regulators p21 and p27 were increased after injection of siSOX2, whereas expression of cyclin-E was decreased, indicating that knockdown of SOX2 suppressed G1/S phase transition (Fig. 6d).

**Fig. 7:**
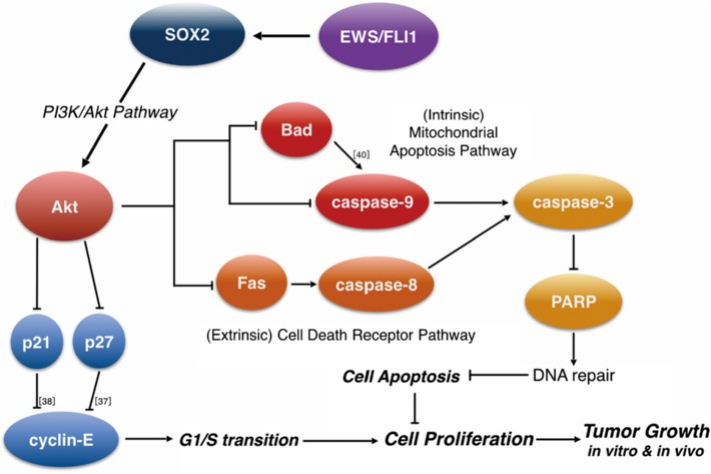
Schematic of the detailed molecular mechanism of SOX2 in regulating cell proliferation of Ewing’s sarcoma in vitro and in vivo

Together, these results verified that SOX2 promoted tumor cell proliferation in Ewing’s sarcoma by restraining apoptosis and promoting cell cycle progression *via* the PI3K/Akt pathway both in vitro and in vivo (Fig. 7).

## Discussion

Ewing’s sarcoma is characteristic with aberrant fusion genes formed by chromosomal translocations, in which *EWS/FLI1* is the most common [4, 5]. SOX2, a key regulator of pluripotency in ESCs, was initially linked to Ewing’s sarcoma by the observation that MSCs transfected with *EWS/FLI1* displayed features of ESFT CSCs [9, 10]. The combination of EWS transactivation domain and the FLI1 DNA-binding domain give rise to heterogeneous breakpoints, the most common being type1 (Exon 7 of EWS/Exon 6 of FLI1) and type2 (Exon 7 of EWS/Exon 5 of FLI1) [4, 6, 32, 46, 47]. These two breakpoints are respectively present in the A673 and RD-ES cell lines used in our study. Consistent with previous reports, we found that silencing *EWS/FLI1* in these cell lines with siRNAs significantly suppressed expression of SOX2, verifying that SOX2 was a downstream target of EWS/FLI1. Furthermore, this study has provided the first evidence that the mechanism involved both the two EWS/FLI1 breakpoints.

In addition to being essential for maintaining cell stemness, SOX2 has been identified as an oncogenic factor in processes associated with tumor progression including cell proliferation and tumorigenesis of ESFT [11]; however, details of the mechanism are poorly understood. Based on our observation that expression of SOX2 was higher in Ewing’s sarcoma tissues compared to normal tissues around the bones, we hypothesized that downregulation of SOX2 might suppress malignant activity in Ewing’s sarcoma.

SOX2 has been reported to facilitate G1/S transition as a mechanism to promote tumor progression in several types of neoplasms, including prostate and breast cancers and some cancers of the digestive system [22–25, 27]. In agreement with these reports, we demonstrated that silencing SOX2 with siRNAs resulted in an accumulation of cells at G0/G1 phase leading to G1/S cell cycle arrest. Further analysis demonstrated that this was mediated through regulation of key cell cycle regulators: cylin-D1 and cyclin-E bind cyclin-dependent kinases (CDKs) to induce transition from G0/G1 to S phase [33, 34]; whereas p21 and p27 act as CDK inhibitors [35, 36]. Our results indicated that SOX2 promoted G1/S transition *via* regulation of cyclin-E, p21 and p27, supporting previous reports that SOX2 plays a central role in tumor cell proliferation in Ewing’s sarcoma.

Previous studies have reported that SOX2 increased apoptotic resistance in several types of malignancies and CSCs [21, 23, 24, 26, 27]. Consistent with these reports, we found that silencing SOX2 resulted in morphologic changes and a significant increase in apoptotic cells in Ewing’s sarcoma. Moreover, the mechanism involved activation of caspase-3 and subsequent amplification of PARP cleavage. PARP plays an important role in DNA repair and can be cleaved by activated capase-3 resulting in cell apoptosis [37].

Further analyses into potential mechanisms revealed that SOX2 possessed regulatory roles in both extrinsic and intrinsic apoptotic pathways. The extrinsic cell death receptor pathway is stimulated by receptors such as Fas and activates caspase-8 [38, 39]; whereas Bcl-2 family proteins are the principle regulators in the intrinsic mitochondrial apoptosis pathway [38, 40]; caspase-9 is pivotal in mitochondrial apoptotic processes and activation of casapse-3 by caspase-8 and caspase-9 initiates cell apoptosis [38]. Our observations confirmed that these actions were replicated in Ewing’s sarcoma: silencing SOX2 promoted apoptosis in Ewing’s sarcoma cells *via* activation of caspase-8 and caspase-9, and stimulated pro-apoptotic factors while repressing anti-apoptotic factors in both the extrinsic and intrinsic pathways.

As a criterion for cell apoptosis assay, however, subG1 phase was not significantly detected in our cell cycle analysis. It could be explained by the reason that DNA fragments were not totally out of the cell membrane and/or subG1 and G0/G1 phases were not sufficiently separated. Similar results were exhibited in some research showing cell apoptosis with no significant subG1 phase under SOX2 silencing [23, 24, 26]. Therefore, we utilized TUNEL and Annexin-V/FITC assays to demonstrate the cell apoptosis, which were much more sufficient and persuasive than subG1 in cell apoptosis investigation.

The role of SOX2 as a transcriptional regulator in critical signaling pathways has been implicated in malignant progressions in various human cancers [16, 19, 20, 23, 25–28]. Of these, the Akt signaling pathway is considered to have the greatest influence on cell cycle progression and apoptosis [42, 43]. Activation of Akt *via* phosphorylation has been shown to directly downregulate pro-apoptotic proteins, such as Bad and caspase-9, indirectly mediate the Fas death receptor [43, 44], and target CDK inhibitors involved in cell cycle regulation, including p21 and p27 [43, 45], thereby promoting cell survival and growth. The present study identified SOX2 as an upstream regulator in the PI3K/Akt pathway in Ewing’s sarcoma; knockdown of SOX2 reduced expression of PI3K and abolished phosphorylation of Akt to p-Akt in vitro; whereas overexpression of Akt counteracted the variations of downstream proteins caused by silencing of SOX2, thereby negating the effects of SOX2 inhibition on the cell cycle progression and apoptosis. However, SOX2 has not been categorically associated with any classical pathway; therefore, it is unclear whether it regulates Akt *via* gene transcription or molecular mediation. In addition, previous studies have reported that SOX2 serves as a downstream target of Akt in various malignancies [16, 19, 23]. So, further investigation is warranted to determine whether loop regulation of SOX2 and Akt exists in Ewing’s sarcoma.

Having verified that SOX2 played significant roles in Ewing’s sarcoma cell proliferation and identified some of the underlying regulatory mechanisms in vitro, it was necessary to confirm these findings in vivo. By establishing Ewing’s sarcoma xenograft models in mice, we were able to demonstrate that treatment with siRNAs against SOX2 could substantially reduce tumor growth of Ewing’s sarcoma in vivo. Furthermore, variations of the factors that had been identified in vitro, including SOX2, regulators of the PI3K/Akt signaling pathway, apoptotic proteins and G1/S transitional mediators, were also verified in xenograft tissue samples.

## Conclusion

In conclusion, our in vitro and in vivo data provided comprehensive evidence that SOX2 played a central role in promoting Ewing’s sarcoma cell survival and proliferation. As a primary target of the *EWS/FLI1* fusion gene characteristic of Ewing’s sarcoma, SOX2 facilitated G1/S phase transition through regulation of key cell cycle factors and restrained cell apoptosis by regulating apoptotic factors in both intrinsic and extrinsic pathways *via* activation of the PI3K/Akt signaling pathway. These findings have identified SOX2 as a potential candidate biomarker for targeted intervention in Ewing’s sarcoma and have provided an experimental basis for future investigations.

### Ethics, consent and permissions

This study was carried out in accordance with the recommendations in the Guide for the Chinese Ethics Review Committees. The protocol was approved by the Ethics Committee of Peking University People’s Hospital. The animal experiment was carried out under ethics approval of Peking University People’s Hospital.

## Abbreviations

SOX2: sex determining region Y-box 2
ESFT: Ewing’s sarcoma family tumor
siRNA: small interfering RNA
siEF: EWS/FLI1 small interfering RNA
siSOX2: SOX2 small interfering RNA
siNC: small interfering RNA negative control
PARP: poly ADP ribose polymerase
CKD: cyclin-dependent kinase
IHC: immunohistochemistry
TUNEL: TdT-mediated dUTP nick end labeling
MSC: mesenchymal stem cells
CSC: cancer stem cells
ESC: embryonic stem cell
